# Modulation of mechanosensory vibrissal responses in the trigeminocervical complex by stimulation of the greater occipital nerve in a rat model of trigeminal neuropathic pain

**DOI:** 10.1186/s10194-020-01161-y

**Published:** 2020-08-06

**Authors:** Nuria García-Magro, Pilar Negredo, Yasmina B. Martin, Ángel Nuñez, Carlos Avendaño

**Affiliations:** 1 Department of Anatomy, Histology and Neuroscience, Medical School, Autonoma University of Madrid, c/ Arzobispo Morcillo 2, 28029 Madrid, Spain; 2 Programme in Neuroscience, Doctoral School, Autonoma University of Madrid, Madrid, Spain; 3 Facultad de Medicina, Universidad Francisco de Vitoria, 28223 Madrid, Spain

**Keywords:** GABA, Glycine, Allodynia, NMDA, Neuromodulation

## Abstract

**Background:**

Stimulation of the occipital or trigeminal nerves has been successfully used to treat chronic refractory neurovascular headaches such as migraine or cluster headache, and painful neuropathies. Convergence of trigeminal and occipital sensory afferents in the ‘trigeminocervical complex’ (TCC) from cutaneous, muscular, dural, and visceral sources is a key mechanism for the input-induced central sensitization that may underlie the altered nociception. Both excitatory (glutamatergic) and inhibitory (GABAergic and glycinergic) mechanisms are involved in modulating nociception in the spinal and medullary dorsal horn neurons, but the mechanisms by which nerve stimulation effects occur are unclear. This study was aimed at investigating the acute effects of electrical stimulation of the greater occipital nerve (GON) on the responses of neurons in the TCC to the mechanical stimulation of the vibrissal pad.

**Methods:**

Adult male Wistar rats were used. Neuronal recordings were obtained in laminae II-IV in the TCC in control, sham and infraorbital chronic constriction injury (CCI-IoN) animals. The GON was isolated and electrically stimulated. Responses to the stimulation of vibrissae by brief air pulses were analyzed before and after GON stimulation. In order to understand the role of the neurotransmitters involved, specific receptor blockers of NMDA (AP-5), GABA_A_ (bicuculline, Bic) and Glycine (strychnine, Str) were applied locally.

**Results:**

GON stimulation produced a facilitation of the response to light facial mechanical stimuli in controls, and an inhibition in CCI-IoN cases. AP-5 reduced responses to GON and vibrissal stimulation and blocked the facilitation of GON on vibrissal responses found in controls. The application of Bic or Str significantly reduced the facilitatory effect of GON stimulation on the response to vibrissal stimulation in controls. However, the opposite effect was found when GABAergic or Glycinergic transmission was prevented in CCI-IoN cases.

**Conclusions:**

GON stimulation modulates the responses of TCC neurons to light mechanical input from the face in opposite directions in controls and under CCI-IoN. This modulation is mediated by GABAergic and Glycinergic mechanisms. These results will help to elucidate the neural mechanisms underlying the effectiveness of nerve stimulation in controlling painful craniofacial disorders, and may be instrumental in identifying new therapeutic targets for their prevention and treatment.

## Introduction

The electrical stimulation of peripheral nerves has become a growing trend during the last decade to treat many drug-resistant painful conditions (recent reviews in [1–3]). Chronic refractory headaches are favored targets for neuromodulation, in particular neurovascular or autonomic-related cephalalgias, such as chronic migraine, cluster headache or hemicrania continua [4–8], and painful neuropathies originating in a trigeminal branch or occipital nerve [9–11]. The stimulation of the greater occipital nerve (GON) has proved successful for medically intractable chronic migraine and neurovascular headaches [12–15], and a few studies have reported promising results in treating refractory trigeminal neuralgia [16].

The mechanisms by which GON stimulation relieves pain from disparate craniofacial territories are still obscure. It is generally assumed that nociceptive input-induced sensitization occurs in second-order neurons on which primary afferents from such territories converge [17, 18], a process that could underlie the development of migraine and trigeminal autonomic cephalalgias [19–21]. Lack of habituation and/or sensitization of central trigeminal neurons have been shown to occur in a variety of primary headaches, both during ictal and interictal periods, and these dysfunctions are a target for the clinical assessment of patients as well as the exploration of novel therapeutic strategies [22–25]. The central node for neural convergence would be the ‘trigeminocervical complex’ (TCC), identified as an area in the upper cervical and medullary dorsal horn where primary afferents from various cutaneous, muscular, dural, and visceral trigeminal and occipital sources distribute [19, 26, 27]. It was first shown in rats that the neurons in the caudal part of the TCC displayed crossed sensitization and functional coupling to input from nociceptive supratentorial dural afferents, which course along the trigeminal nerve, and cervical afferents conveyed through the GON [19, 28]. However, in a rat model of migraine GON electrostimulation induced lasting elevations of mechanical allodynia thresholds at trigeminal and other bodily regions [29], and reduced tonic and burst firing of ventroposteromedial thalamic neurons in response to mechanosensory stimuli [30].

In an attempt to clarify these apparently conflicting results, Lyubashina et al. [31] studied the effect of GON preconditioning using different stimulation parameters on eleven TCC neurons that showed convergence of GON, supratentorial dura and facial cutaneous inputs, and found consistent reductions in the spontaneous activity and responsivity to receptive field stimulation. These authors suggested that convergence on wide-dynamic range (WDR) neurons in TCC could result in facilitation or inhibition, depending on the stimulation parameters and the nociceptive state of the subject. In fact, both excitatory glutamatergic and inhibitory GABAergic mechanisms have been involved in the modulation of nociception in TCC neurons. The NMDA receptor blockade reduced the increase in c-Fos expression in superficial laminae of TCC induced by electrical or chemical stimulation of the dural sinuses or the occipital muscles [32, 33], and inhibited nociceptive perivascular dural stimuli-induced firing in laminae I-IV neurons of spinal segments C_1_-C_2_ [34, 35]. Moreover, following the demonstration that GABA modulates nociceptive input in the TCC through GABA_A_ receptors [36], it was shown that pain-generating peripheral nerve injuries resulted in GABA molecular markers and GABA-mediated inhibition in the dorsal horn being reduced, both in spinal cord slices [37, 38], and in the caudal division of the spinal trigeminal nucleus (Sp5C) in anesthetized rats [39].

In this study we aimed at investigating the acute effects of the electrical stimulation of the greater occipital nerve (GON) on the responses of neurons in the TCC to the mechanical stimulation of the vibrissal pad in control rats and rats that display allodynia following constriction injury of a trigeminal nerve branch. Our results found modulating and opposite effects of GON stimulation in each group and shed light on the involvement in these effects of NMDA-dependent excitatory and GABAergic and Glycinergic inhibitory mechanisms.

## Materials and methods

### Animals and IoN surgery

Forty-three 3-month-old male Wistar rats (RccHan:Wis, ENVIGO, The Netherlands) were used in this study. All procedures followed the regulations issued by the Ethical Committee of the Autonoma University of Madrid and the European Community’s Council Directive 2010/63/UE. All efforts were made to reduce the number of animals used and their suffering.

Chronic infraorbital nerve constriction (CCI-IoN) was performed on the right side of the animal to induce trigeminal neuralgia [40]. Animals were anesthetized by intramuscular injection of Ketamine (Ketolar, 55 mg/kg), Xylazine (Rompun, 15 mg/kg) and Atropine (0.2 g/kg). The IoN was exposed under the vibrissal pad and a single polypropylene monofilament (Surgipro 6.0) ligature was loosely tied around the distal part of the nerve. This procedure altered little, if at all, the circulation through the superficial epineural vasculature [41, 42]. Sham-operated control rats underwent the same surgical procedure until exposing the nerve, which was left untouched. The facial wound was closed with interrupted silk sutures. Age-matched control rats were not operated. After recovery from surgery the operated animals’ behavior during grooming or eating did not differ from the controls.

### Behavioral testing

The evaluation of mechanical allodynia was performed using a series of calibrated von Frey nylon monofilaments (North Coast Medical, Inc., Morgan Hill, CA, USA). For 2 days before starting the behavioral tests, the animals were habituated for 1 h daily to the environment (a quiet room with low red lighting) and the experimenter. Daily tests were then performed for three consecutive days before surgery, to establish the baseline response score, and on days 7, 14 and 21 after surgery. Tests consisted of the application of von Frey’s filaments to the bending point on different points of the vibrissal pad. Each filament was presented in three series of five times at 10–20 s intervals. The first series started randomly on the left or right pad. In the first few control and CCI-IoN rats six filaments, between 0.07 and 8.0 g, were presented in a sequential ascending order. Since the responses to the thinner filaments failed to evoke significant responses in either group, only 4.0 g and 8.0 g filaments were tried on the remaining animals. A reduction in the number of filaments used also has the advantage of reducing the testing time and possible stress to the animal [40, 43]. The responses recorded (and value assigned) were: Face withdrawal (0.25 points), brisk head shaking (0.5 points), face withdrawal + ipsilateral eye blink (1.0 point), vocalization (1.0 point) and vigorous face scratching (1.5 points). The points were added up for each testing time point and averaged over the two filaments used to give an overall ‘response score’.

### Electrophysiological recordings and stimulation

One day after the final behavioral testing session (22 days post-surgery, dps) in cases with CCI-IoN), the animals were anesthetized with urethane (1.6 g / kg i.p.) to perform unit recordings. With the head of the animal positioned in a stereotactic frame (David Kopf Instruments), a midline incision was made in the skin over the occipital and upper cervical levels. The skin was retracted and secured laterally, together with the cervical muscles, to expose the GON and the first two vertebrae. The GON was identified through the thin layers of fascia [27], and 2 mm of the nerve were gently separated from the muscle and connective tissue. The posterior arch of the atlas was removed and the atlanto-occipital membrane and the underlying dura mater were incised to expose the caudal part of Sp5C and the spinal segment C_1_.

Tungsten microelectrodes (2 MΩ, World Precision Instruments) were lowered with a micromanipulator obliquely into the brain stem, to obtain single unit recordings from neurons in laminae II-IV of the right TCC. The vibrissal pad was mechanically stimulated with electronically gated 20 ms air puffs through a thin (1 mm inner diameter) polyethylene tube placed at 5–7 mm distance from the skin, and at a constant pressure (3 psi) using a Picospritzer II. The GON was electrically stimulated with 0.2 ms single pulses delivered by a Cibertec Stimulator (Madrid, Spain) using a monopolar electrode (150 μm, blunt cut stainless steel wire) placed on the GON. The current applied ranged between 100 and 400 μA, ensuring the absence of muscle twitches.

The experimental protocol consisted of a 9 s period of spontaneous activity, followed by 50 air puffs delivered at 0.3 Hz. After that, 50 GON pulses at 0.3 Hz were delivered; each was followed 100 ms later by an air puff on the vibrissal pad. Signals from TCC recordings were filtered (0.3–3 kHz) and fed to a personal computer at 10 kHz sampling rate with stimuli events for off-line analysis with Spike 2 (Cambridge Electronic Design, Cambridge, UK). In most cases, small electrolytic lesions (1–2 μA DC for 10 s) were made with the same electrode at the end of the session to mark the recording site.

### Drug application

The global pharmacological blockade of NMDA, GABA and/or Glycine receptors in the TCC, particularly in its superficial laminae, was attempted by local infusion of specific receptor antagonists. Amino-5-phosphonovaleric acid or (AP-5, a NMDA receptor antagonist, 50 μM), Bicuculline methiodide (Bic, a GABA_A_ receptor antagonist, 20 mM), Strychnine (Str, an antagonist of the Glycine receptor, 100 μM), or a mixture of Str and Bic were mechanically delivered over the TCC through a glass micropipette (20–30 μm tip outer diameter) attached to a 10 μl Hamilton syringe. The injected volume was 2 μl. All drugs (Sigma, St Louis, MO, USA) were dissolved in saline solution (0.9% NaCl). Recordings were started immediately after drug application, with the same protocol as before.

### Tracer injections

In two control rats, the right trigeminal ganglion (TG) was injected with a transganglionic tracer to appraise the area of overlap of primary afferents from the trigeminal nerve and the GON. Data for the latter were recovered from material used in a prior study from our group (García-Magro et al., 2018). Briefly, the rats were anesthetized with an i.m. injection of Ketamine (Ketolar, 55 mg/kg), Xylazine (Rompun, 15 mg/kg) and Atropine (0.2 g/kg). The TG was directly accessed through a lateral craniotomy and gentle displacement of the ventral part of the hemisphere. Using a glass micropipette (Systems, Inc) coupled to a 10 μl Hamilton syringe, 2 μl of a 10% biotinylated dextran amine (BDA 3000 Invitrogen-Molecular, Eugene, OR, USA) solution in saline were slowly injected into Meckel’s cavum. Other animals received an intraneural deposit of a mixture of 1% cholera toxin B (CTB, Sigma-Aldrich) with 2% isolectin IB4 from *Griffonia simplicifolia* (Vector Laboratories) into the right GON. Postinjection survival times were 10 and 4 days for rats injected in TG and GON, respectively. The animals were then deeply anesthetized (Dolethal, 50 mg / kg i.p.) and perfused through the ascending aorta with 4% paraformaldehyde (PFA) in 0.1 M phosphate buffer (PB). The upper cervical spinal cord and caudal two-thirds of the brain stem were extracted, postfixed in the same fixative overnight at 4 °C and cryoprotected with a 30% sucrose solution in 0.1 M PB for 2 days.

### Tissue processing and staining

The blocks selected for immunohistochemistry were frozen and cut at 40 μm in the horizontal plane using a sliding microtome (Leica SM2400, Leica Biosystems, Nussloch). All sections were processed free-floating. Series of sections from TG-injected cases were incubated in avidin–biotin peroxidase (Kit ABC Elite®, 1:250 in PBS; Vector Laboratories, Burlingame, CA, USA) and revealed with diaminobenzidine (DAB, 0.05% in PBS; Sigma, St. Louis, MO, USA) adding 0.001% H_2_O_2_. Series from GON-injected rats were first incubated in rabbit anti-CTB (1:500; Sigma Aldrich) or goat anti-IB4 (1:1000; Vector Laboratories) followed by biotinylated goat anti-rabbit (1:500; Sigma Aldrich) or rabbit anti-goat (1:250; Vector Laboratories), and then treated in the same way. All sections were mounted on glass slides, dehydrated, defatted and coverslipped with DePeX.

Blocks containing the recording regions were frozen and cut at 40 μm in the coronal plane. Sections were Nissl-stained (0.25% cresyl violet) to visualize electrode tracks and electrolytic lesion marks.

Material prepared for a previous study [44] was recovered to identify the GABA and Glycine immunoreactive cells in the TCC. Briefly, small blocks containing the medullary dorsal horn were trimmed from resin-embedded slices and serially sectioned at 1 μm using an ultramicrotome. Pairs of adjacent sections were collected, and mounted on separate glass slides, which were etched, osmicated and incubated in a wet chamber with either monoclonal anti-GABA antibody (1:250; clone 3D5, [45]), or polyclonal anti-glycine antiserum raised in rabbit (1:1000; Ab139, Chemicon Europe, Hampshire, UK). Appropriate biotinylated secondary antibodies were then used, followed by incubation in ABC and DAB as above.

### Data analysis

Units were accepted for statistical analysis when the fluctuations of the unit amplitude were lower than 10% over the course of the experiment and were also large enough to be well-isolated from multiunit firing activity. Single-unit activity was discriminated by threshold spike detection using SPIKE 2 software for the offline spike sorting (Cambridge Electronic Design, Cambridge). Peristimulus time histograms (PSTHs; 1 ms bin) of neural responses were also analyzed using SPIKE 2 software. The response was analyzed during the period of vibrissal stimulation and compared with the following period in which GON stimulation had been paired with vibrissal stimulation (100 ms delay). The spike response was measured from the PSTH as the number of spikes evoked in the 0–50-ms time window after the stimulus onset, divided by the number of stimuli. Response latency was defined as the time elapsed between stimulus onset and the largest peak in the PSTH.

### Statistical analysis

Descriptive statistics (means and SEM) for the parameters analyzed were obtained from the Excel spreadsheet used to perform calculations (Microsoft Office Professional Plus 2010 for Windows 10). Any differences between variables were compared using two-way parametric (Student’s t test) or non-parametric (Wilcoxon) tests, after normality testing (D’Agostino-Pearson), with GraphPad Prism software (v. 8.0 for Windows). For behavioral analysis data, global comparisons between the groups and days post-injury were made by two-way ANOVA with repeated measures and SPSS (v. 15). The level of significance was given by the *P*-value and was represented in Figures as * (*p* < 0.05), ** (*p* < 0.01) and *** (*p* < 0.001).

## Results

### Coincidence of GON and TG afferents in the TCC

**Fig. 1:**
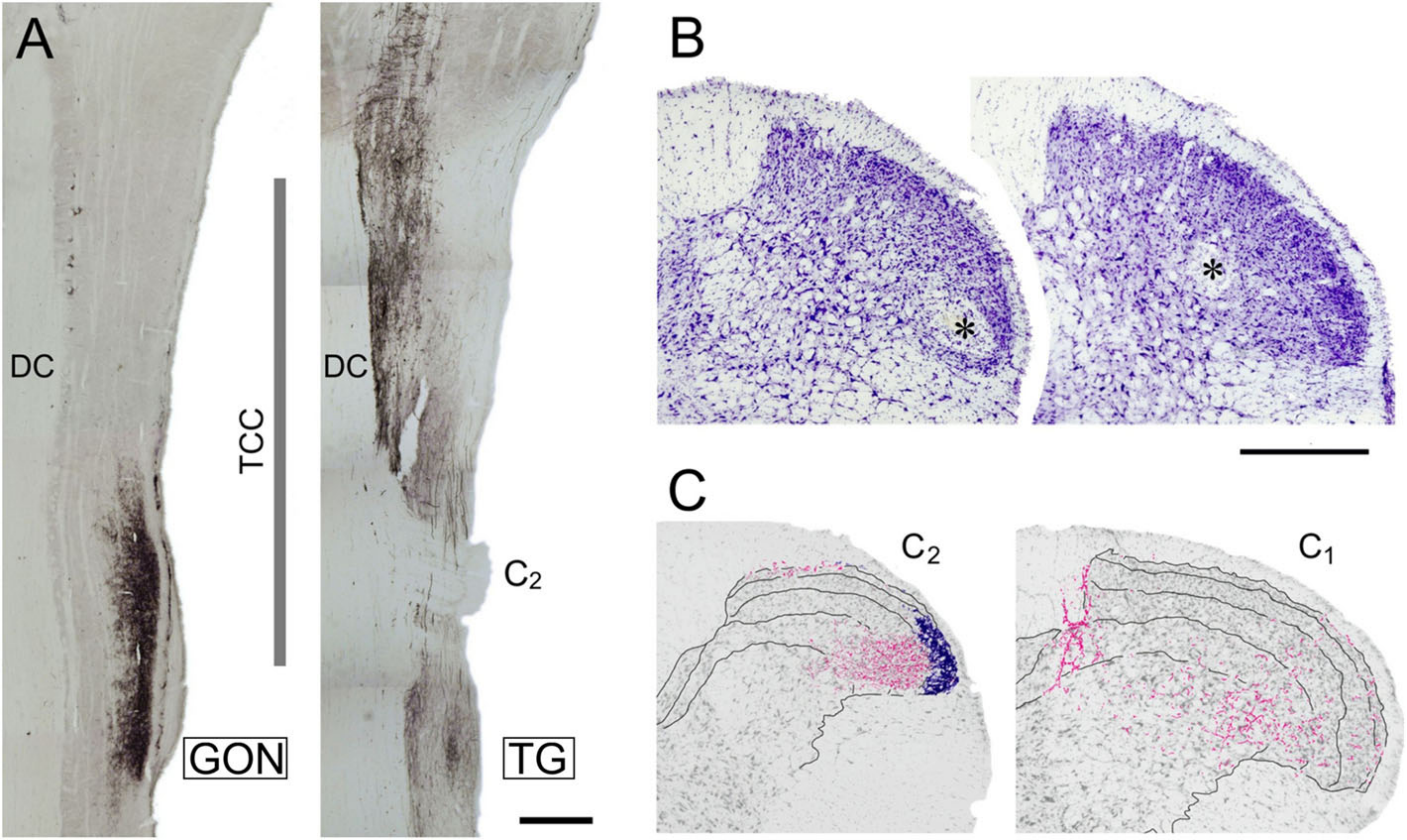
(**a**) Coincidence of primary afferents from the GON (left) and the TG (right) in the TCC region. CTB-immunolabeled fibers are shown in two horizontal sections through dorsal levels of spinal and medullary regions that extend from mid-medullary levels (top) to cervical segment C_3_ (bottom). The vertical line marks the approximate rostrocaudal extent of the TCC. In these sections it is clearly seen that afferents from the GON concentrate in TCC at the level of segment C2, while TG afferents extend continuously further caudally. The seeming interruption in TG labeling corresponds to the entry of the dorsal root of spinal nerve C_2_ entering the cord, selected for topographic reference. DC, dorsal column; TCC, trigeminocervical complex. Scale bar = 500 μm. (**b**) Two representative examples of small electrolytic lesions (asterisks) through the recording electrode at the end of the recording session. These cases show recording sites in laminae II inner (left) and IV (right) in the ventrolateral one-half of the dorsal horn. Most recordings were placed within the area demarcated by these lesions. (**c**) The recording sites fell within a territory with substantial innervation of CTB-labeled fibers from the GON (shown in magenta). I-IV laminar boundaries are outlined, and blue stippling indicate IB4-labeled, presumably unmyelinated, afferents in laminae I and II, which were virtually restricted to segments C_2_ and C_3_ (diagram reproduced from Garcia-Magro et al., 2018). Scale bar = 500 μm

CTB-labeled, putatively myelinated, afferents from the GON profusely innervate laminae I and III-V of the lateral dorsal horn of the first 6 cervical spinal segments. Sparser projections extend into neighboring Sp5C. IB4-labeled, putatively unmyelinated afferents, distribute exclusively in laminae I-II of the lateral dorsal horn of segments C_2–3_ (Fig. 1; see also [27]). Abundant BDA-labeled primary afferents from the TG densely innervate the trigeminal nuclei, reaching the dorsal horn of the upper half of C_2_ and, more sparsely, further caudally in the spinal cord (Fig. 1a). Recordings from the TCC were obtained from laminae II (inner part), III, or superficial part of IV in the ventrolateral half of the dorsal horn between the caudal part of Sp5C and the upper part of C_2_ (Fig. 1b, c).

### CCI-IoN induced consistent mechanical allodynia

**Fig. 2:**
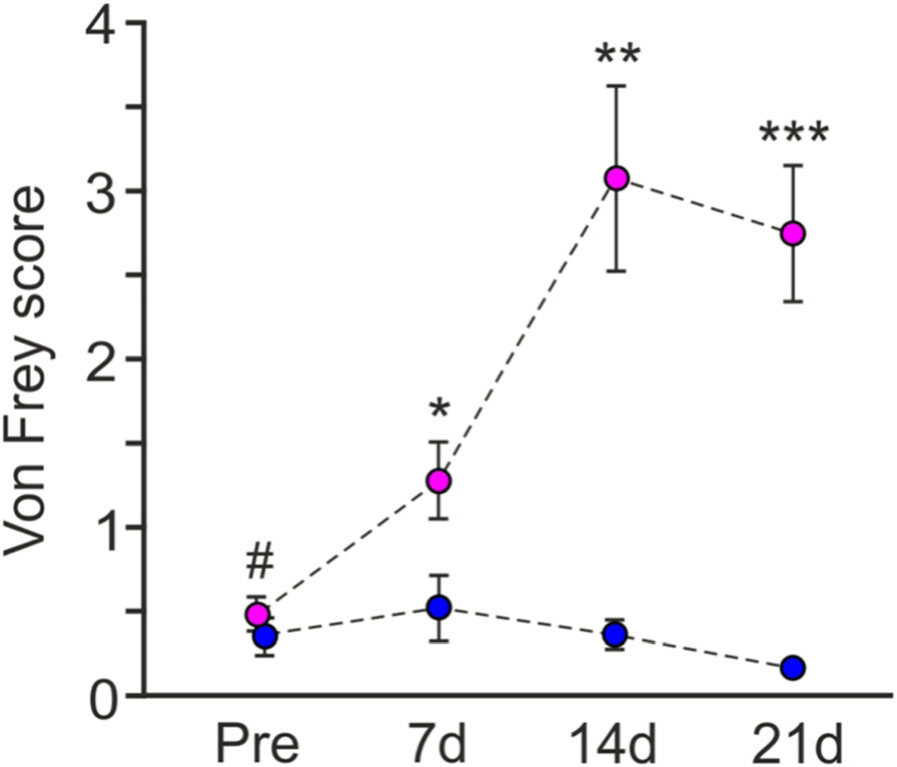
Testing withdrawal responses to whisker pad mechanical stimulation with von Frey filaments shows a distinct time course over 3 weeks following unilateral (right side) CCI-IoN. The response score differed significantly between sham-operated (blue; *n* = 10) and CCI-IoN (magenta; *n* = 16) groups regarding Group (F_1,107_ = 28.906, *p* < 0.001) and Time (F_3,6_ = 4.217, *p* = 0.007), and also showed significant Group x Time interaction (F_3,107_ = 5.228, *p* = 0.002). Data represent means ± SEM, two-way ANOVA with repeated measures, Dunnett T3 post hoc test. Asterisks correspond to *p* < 0.05 (*), *p* < 0.01 (**), and *p* < 0.001 (***) for between-group comparisons. Within-group comparisons showed no changes in control cases, but a significant increase from baseline, pre-CCI values (#), over the three postsurgery testing days (*p* = 0.010, 0.003 and 0.001 for 7, 14, and 21 dps, respectively)

The responses to mechanical stimuli were evaluated at 7, 14 and 21 dps, and compared with the mean control values during the 3 days prior to surgery. As described previously [39, 40], after CCI-IoN the rats developed hypersensitivity to mechanical stimulation on the side ipsilateral to the lesion. The CCI-IoN animals showed a moderate but significant increase on day 7 (*p* = 0.010) that reached a maximum on postlesion day 14 (*p* = 0.003) and persisted until the last testing day (*p* = 0.001) (Fig. 2). This increase in responses was restricted to the vibrissal pad, the main territory innervated by the IoN. Responses on the contralateral side were similar to those in control animals, except for some cases that also developed contralateral allodynia, although this was less pronounced than in the operated side (data not shown).

### TCC neurons respond differently to vibrissal and GON stimulation under control or CCI-IoN conditions

**Fig. 3:**
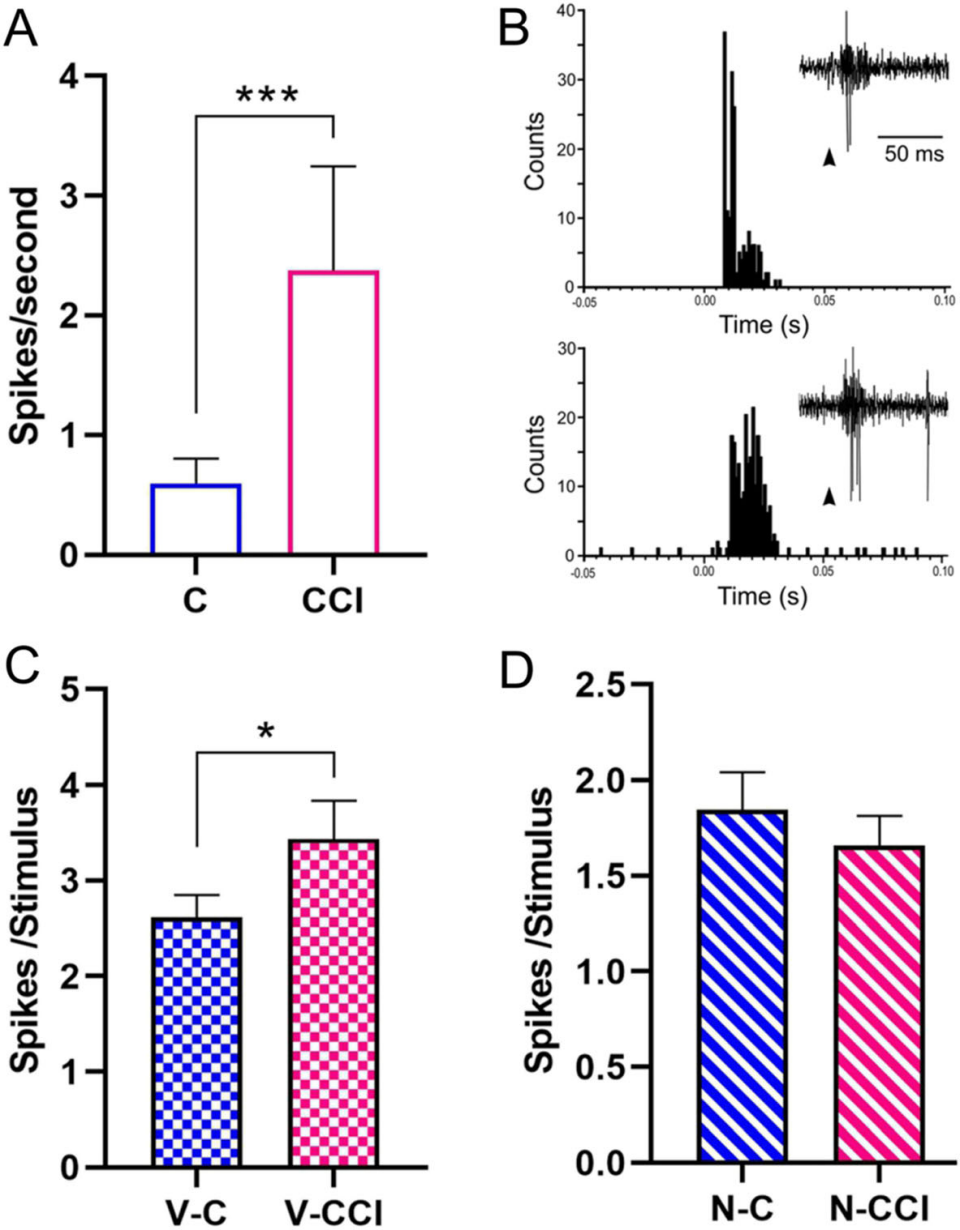
Changes in spontaneous and input-evoked activity of TCC neurons after CCI-IoN. (**a**) Frequency of spontaneous discharges increased four-fold in CCI-IoN animals (C, control group; CCI, CCI-IoN group). (**b**) Representative PSTHs of vibrissal responses (50 stimuli) in a control case (upper plot) and in a CCI-IoN rat (lower plot). Insets show raw data examples; arrowheads indicate stimulus onset. The response was higher in the CCI-IoN rat. (**c**) Plot of the mean vibrissal response in control and in CCI-IoN animals. The response was greater in CCI-IoN animals. (**d**) The response to GON stimulation did not differ significantly between control and CCI-IoN animals. V-C, vibrissal response in control animals; V-CCI, vibrissal response in CCI-IoN animals; N-C, GON response in control animals; N-CCI, GON response in CCI-IoN animals; * *p* < 0.05; *** *p* < 0.0001

Spontaneous and evoked activity was examined in TCC neurons in control animals and in the CCI-IoN animals at 22 dps, when allodynia was well established. All neurons displayed an ipsilateral RF restricted to a small caudal part of the vibrissal pad, corresponding to one or two adjacent vibrissae in arcs 1–2 or neighboring straddlers [46]. The TCC neurons did not fire, or displayed a low mean firing rate, under spontaneous conditions in control animals (0.6 ± 0.2 spikes/s, *n* = 49 neurons, Fig. 3a). Following CCI-IoN, the spontaneous activity of TCC neurons increased fourfold (2.4 ± 0.9 spikes/s, *n* = 27 neurons, *p* < 0.0005 unpaired test).

Tactile stimuli deflecting the vibrissae (20 ms duration; Fig. 3b) evoked 2.6 ± 0.23 spikes/stimulus (*n* = 49) in control animals (measured between 0 and 50 ms after the stimulus onset; see Methods) with a mean latency of 8.4 ± 0.3 ms. This response was a 30% higher in CCI-IoN animals (3.4 ± 0.4 spikes/stimulus; *n* = 27, *p* = 0.0307 unpaired test; Fig. 3c), without a change in latency with respect to controls (8.6 ± 0.2 ms).

Single electrical stimuli (0.2 ms duration) applied to the GON consistently elicited a response in TCC neurons of 1.8 ± 0.2 spikes/stimulus (*n* = 49) in the control animals (measured between 0 and 50 ms after the stimulus onset) with a mean latency of 3.4 ± 0.3 ms. In the CCI-IoN animals, their responses to GON stimulation were similar (1.7 ± 0.15 spikes/stimulus; *n* = 27 neurons, *p* = 0.767 unpaired test; Fig. 3d), as was the response latency (3.3 ± 0.3 ms).

### GON stimulation modulates vibrissal responses in TCC

**Fig. 4:**
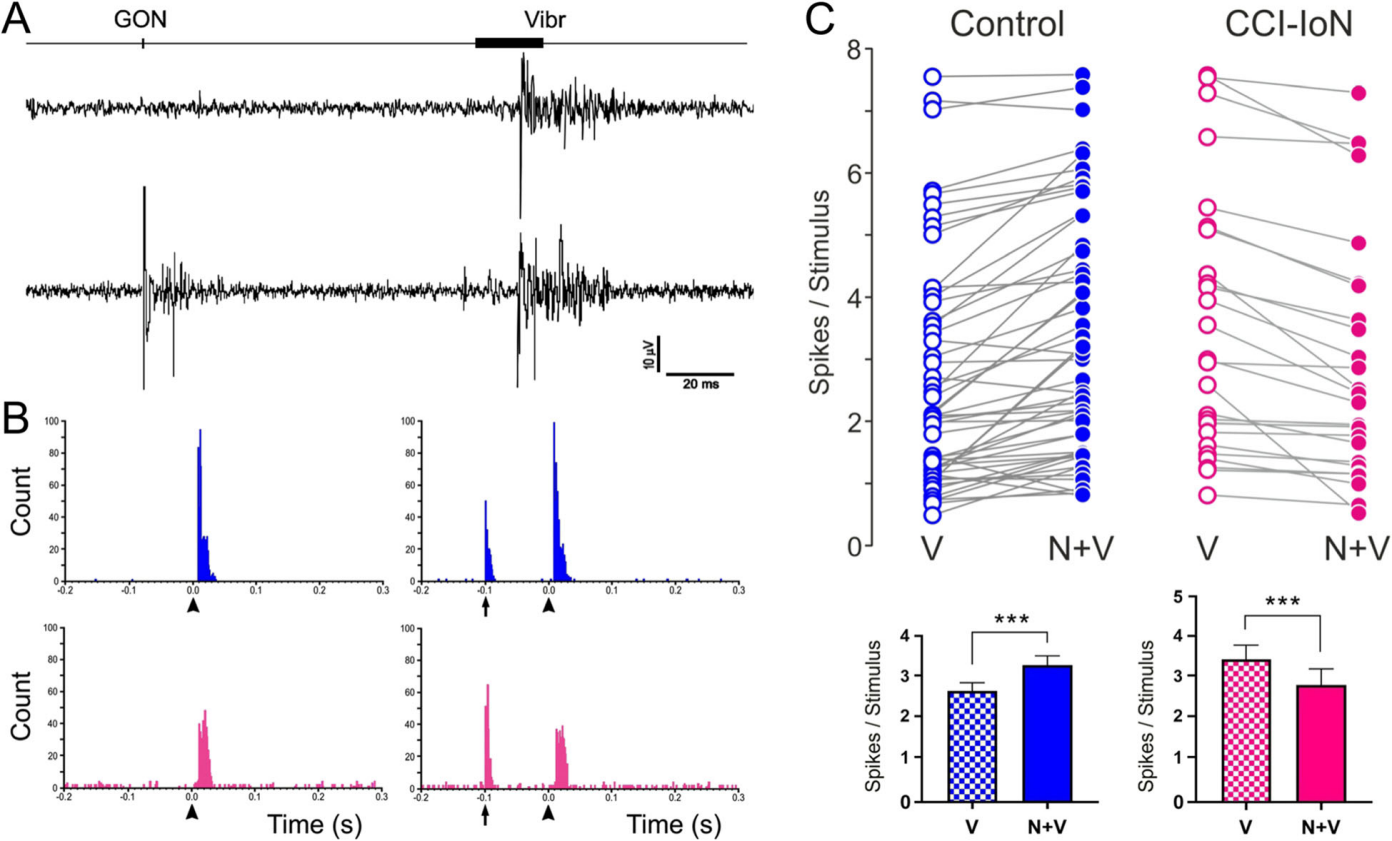
Effect of GON stimulation on vibrissal responses in control (blue) and CCI-IoN (magenta) animals. (**a**) An example of raw recordings during vibrissal stimulation alone (upper trace) or preceded by GON stimulation (lower trace). (**b**) Representative PSTHs in a control case (upper plot) and a CCI-IoN case (lower plot). Plots on the left show the response to vibrissal stimulation (arrowheads) alone. On the right, responses to vibrissal stimulation (arrowheads) when preceded by GON stimulation (vertical arrow) are illustrated. In the control case (top) GON stimulation facilitated vibrissal response, whereas the opposite occurred in the CCI-IoN. (**c**) Top, same-case comparisons between vibrissal responses alone (V, empty circles) and when preceded by GON stimulation (N + V, 100 ms delay; filled circles) in control (blue traces) and CCI-IoN animals (red traces). Bottom, mean (+sem) vibrissal responses from the data above. GON stimulation facilitated vibrissal response in control animals but inhibited vibrissal responses in CCI-IoN animals. *** *p* < 0.0001

The GON was electrically stimulated (1 pulse; 0.2 ms) at different delays before vibrissal stimulation to study its effect on vibrissal responses in the control condition and when allodynia was established in the CCI-IoN animals. The average effect was a facilitation of vibrissal responses in control animals when the delay between the stimuli was shorter than 300 ms. For performing comparisons, we chose a 100 ms interval (Fig. 4a, b). GON stimulation increased vibrissal responses from 2.6 ± 0.23 spikes/stimulus to 3.3 ± 0.24 spikes/stimulus when GON and vibrissal stimulation were, paired (*n* = 61; *p* < 0.0001, paired test; Fig. 4c). This effect was observed in 55 of the 61 TCC cells; in 3 neurons GON stimulation did not modify the response and the response decreased in 3 cells. However, the effect of GON stimulation changed to an inhibition of vibrissal responses when GON and vibrissal stimulation were paired in CCI-IoN animals (from 3.4 ± 0.4 spikes/stimulus in control to 2.8 ± 0.38 spikes/stimulus with GON stimulation; *n* = 27; *p* < 0.0001, paired test). This effect was observed in 22 of the 27 TCC cells; in the remaining 5 neurons GON stimulation did not modify the response. The response latency was not modified in any case by the stimulation of the GON (data not shown).

### GON-evoked facilitation is dependent of NMDA receptors

**Fig. 5:**
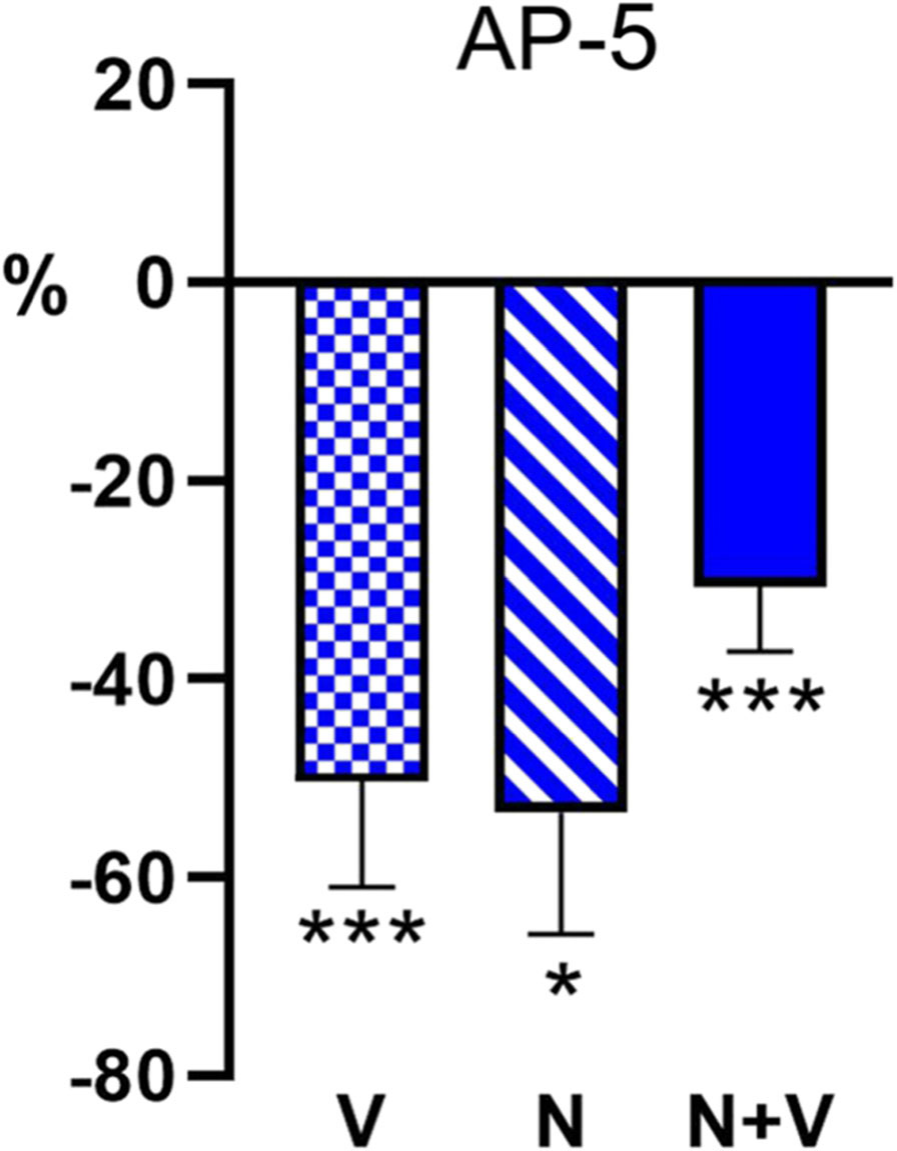
Blocking of NMDA receptors by AP-5 caused a significant decrease in TCC neuronal responses. Vibrissal (V) and GON (N) responses were halved by AP-5 (50 μM). In addition, the GON-evoked facilitation was blocked by AP-5 and unmasked a GON-evoked inhibition. * *p* < 0.05; *** *p* < 0.0001. All values are expressed as percentages with respect to the corresponding basal responses before AP-5 application

Vibrissal responses in TCC neurons were reduced when the NMDA-receptor antagonist AP-5 was applied on the TCC area in 10 out of 12 neurons. The response was reduced by 50.2 ± 10.8% 10 min after AP-5 application (*n* = 12; *p* = 0.0034, paired test; Fig. 5). Although previously unreported, we found that AP-5 application also reduced the response of TCC neurons to GON stimulation by 53.5 ± 12.3% 10 min after AP-5 application in all the cells tested (*n* = 7; *p* = 0.0156, paired test). Accordingly, the GON-evoked facilitation of vibrissal responses observed in the control animals was blocked after AP-5 application (by 30.7 ± 6.5%; *n* = 12; *p* = 0.005, paired test).

### Inhibition may modulate TCC neurons in control and in CCI-IoN animals

**Fig. 6:**
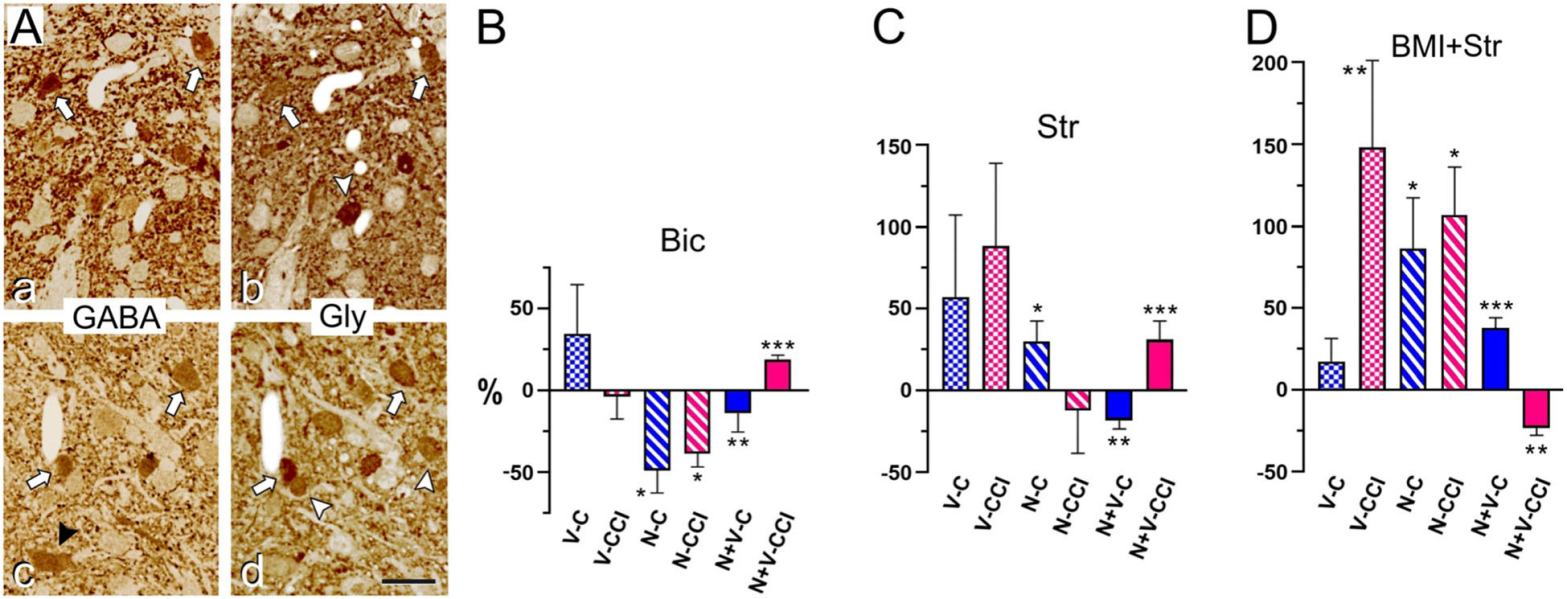
(**A**) Examples of two pairs of GABA- (**a**, **c**) and Glycine-immunoreacted (**b**, **d**) consecutive semithin sections from laminae I-II (**a**, **b**) and III (**c,d**) at rostral levels of the TCC in a control rat. In agreement with previous findings (Avendaño et al., 2005), over one-third of neuron profiles display immunoreactivity for either GABA or Glycine. The majority shows various degrees of coexpression (some are indicated by white arrows), followed by those expressing only Glycine (white arrowheads). A small number of cells are only GABA-immunolabeled (black arrowhead in **c**). Scale bar = 20 μm. (**B**-**D**) Effect of antagonists of GABA- and Glycinergic neurotransmission on TCC neuronal responses. (**B**) In presence of the GABA_A_ receptor antagonist bicuculline (Bic; 20 mM), GON responses were reduced both in controls and CCI-IoN cases, whereas vibrissal responses were essentially unaltered. (**C**) By contrast, the Glycinergic receptor antagonist strychnine (Str; 100 μM) increased, albeit non-significantly, the vibrissal responses in both groups, and significantly increased GON responses in controls. (**D**) When both antagonists were applied simultaneously (i.e. the ‘cocktail’ application; Bic, 20 mM + Str, 100 μM) there was a general increase of responses to vibrissae and GON stimulation, which only failed to reach significance for vibrissal responses in controls. The interaction of GON and vibrissal stimulation was affected in all cases by Bic, Str, or the ‘cocktail’ application, in the same directions if any of the drugs had been applied separately (reduced response to vibrissae in controls, and enhanced in CCI-IoN cases), and in opposite directions when both drugs were applied simultaneously. Abbreviations as in Figs. 3 and 4. All values are expressed as a percentage of change with respect to the corresponding basal responses before each drug application. *, *p* < 0.05; **, *p* < 0.01; ***, *p* < 0.001

Vibrissal and GON stimulation induced excitatory responses in TCC neurons, which are in turn controlled by local inhibitory interneurons, which express GABA, Glycine, or both transmitters simultaneously (Fig. 6A; [39, 44]).

#### Effects of GABA_A_ receptor blockade

Contrary to expectations, Bic application did not show a significant change in the spontaneous activity in 11 of the 13 neurons in control animals (from 0.6 ± 0.46 spikes/s in basal condition to 0.2 ± 0.19 spikes/s after Bic; *n* = 13; *p* = 0.75, paired test), nor in CCI-IoN cases (from 3.7 ± 1.49 to 3.42 ± 1.19; *n* = 15; *p* = 0.96). Moreover, the vibrissal responses decreased in 5 of the 13 neurons, increased in 7 neurons and in 1 they remained unchanged, resulting in a non-significant 34.4 ± 30.1% increase after Bic (*n* = 13; *p* > 0.9, paired test; Fig. 6B). GON-responses decreased in the majority of neurons (7 out of 8) after Bic (by 49.62 ± 13.6%; *n* = 8; *p* = 0.023, paired test) in controls. In the CCI-IoN cases, Bic had little effect on the responses to vibrissal stimulation: they decreased in 9 of the 15 neurons, increased in 4 neurons and remained unchanged in 2 neurons, resulting in a non-significant 4.13 ± 13.3% decrease in the whole population (*n* = 15; *p* = 0.56, paired test; Fig. 6B). As in the control cases, GON-responses were also significantly reduced by Bic in the CCI-IoN animals: all the neurons tested showed a decrease, which reached on average 39.14 ± 7.8% (*n* = 7; *p* = 0.015, paired test). GON stimulation inhibited vibrissal responses in the presence of Bic (by 14.3 ± 11.2%; *n* = 13; *p* = 0.008, paired test). Conversely, in the CCI-IoN animals, GON stimulation induced a facilitation of the vibrissal response after Bic application (by 18.6 ± 2.7%; *n* = 15; *p* < 0.0001, paired test).

#### Effects of Glycine receptor blockade

Str application increased spontaneous firing in 9 of the 10 TCC neurons in controls (from 0.1 ± 0.09 spikes/s in basal condition to 1.0 ± 0.51 spikes/s after Str application; *n* = 10; *p* = 0.008, paired test), and –although not significantly- in 6 of the 12 neurons in CCI-IoN cases (from 0.7 ± 0.24 spikes/s in basal condition to 1.7 ± 0.53 spikes/s under Str; *n* = 12; *p* = 0.080, paired test). Responses to vibrissal stimulation varied considerably in the TCC, with 4 of the 10 neurons increasing their response, 4 decreasing and 2 remaining unchanged (resulting in a non-significant 57.1 ± 50.3% mean increase; *n* = 10; *p* = 0.65, paired test; Fig. 6C). In contrast to Bic, Str increased GON-responses in all the neurons tested (by 30.2 ± 12.5%; *n* = 7; *p* = 0.031, paired test). In the CCI-IoN animals, Str also resulted in a variable response pattern to vibrissal stimulation: 6 of the 12 neurons increased their response, 5 decreased their response and in one of the neurons no effect was observed. This apparently large increase in responsivity (by 89.1 ± 50%) failed to reach statistical significance (*n* = 12; *p* = 0.73, paired test). GON-responses in the CCI-IoN animals showed a non-significant decrease in response in 6 of the 7 neurons (by 15.57 ± 21.9%; *n* = 7 *p* = 0.43, paired test; Fig. 6C). After Str application, GON stimulation inhibited vibrissal responses (by 18.6 ± 5.3%; *n* = 10; *p* = 0.008, paired test). By contrast, in the CCI-IoN animals, GON stimulation induced a facilitation of the vibrissal response after Str application (by 31.3 ± 11.13%; *n* = 12; *p* < 0.001, paired test).

#### Effects of combined GABA_A_ and Glycine receptor blockade

The application of a ‘cocktail’ containing Bic and Str (Fig. 6D) increased GON-responses in all cells tested (by 66.8 ± 20.03%; *n* = 5; *p* = 0.03, paired test). In concurrence with above results, the application of the ‘cocktail’ did not significantly affect the vibrissal responses in the control TCC neuronal population, which slightly increased in 8 of the 12 neurons (by 17.9 ± 13.4%; *n* = 12; *p* = 0.42, paired test). This treatment in the CCI-IoN animals, however, induced a significant increase of vibrissal response in the neuronal population (by 139.5 ± 33.5%; *n* = 10; *p* = 0.002, paired test), as well as of their responses to GON stimulation (by 127.1 ± 45.3%; *n* = 5 *p* = 0.03, paired test). Compared with the effects of separate Bic or Str applications, when both blockers were applied GON stimulation exerted the opposite effects on the vibrissal response in all the TCC neurons. In the controls, the response increased by 38.1 ± 6.04% (*n* = 12; *p* < 0.001, paired test), whereas in the CCI-IoN animals their responses decreased by 24.16 ± 4.4% (*n* = 10; *p* = 0.002, paired test).

## Discussion

The present results confirm and extend previous evidence of a convergence of trigeminal and GON afferents in the TCC (reviewed in [26]), and show that GON stimulation exerts different modulatory effects on trigeminal input in the TCC of intact animals and in animals that display pain and allodynia induced by the CCI of the IoN. When paired at intervals < 300 ms, GON stimulation facilitates, in the TCC, neuronal responses to subsequent innocuous tactile stimuli to the vibrissal pad in control cases, whereas the same stimulation evokes an inhibition of vibrissal responses in CCI-IoN animals, an effect that is mediated by GABAergic and Glycinergic mechanisms. Vibrissal and GON stimulation-evoked spike firing of TCC neurons involve NMDA glutamatergic receptor activation, since it was reduced by the application of a NMDA-receptor antagonist, as also shown for responses to occipital muscle input in the TCC [33]. These findings may help to explain the beneficial effects of GON stimulation in treating some refractory craniofacial pain syndromes that involve trigeminal territories [16, 47, 48].

### Primary afferents putatively driving the responses observed in TCC

Impulses elicited by mild mechanical stimuli applied to the whisker pad are likely to emerge from all the mystacial, non-mystacial and intervibrissal fur low-threshold mechanoreceptors (LTMR) innervated by thickly- (Aβ) and thinly-myelinated (Aδ) fibers [49–52]. In the spinal cord, Aβ fibers distribute mainly in lamina III, with terminal and *en passant* boutons. Most boutons form the central component of type IIb glomeruli [53]), receiving axo-axonic contacts from presynaptic axons that express both GABA and Glycine (55–75%, depending on the type of afferent), GABA only (25–40%), or only Glycine (0–10%), and being in turn presynaptic to dendrites, which only in a small fraction of cases express either or both of these inhibitory transmitters [54–57]. In deeper laminae afferent boutons from Aβ fibers are replaced by simpler boutons, many of which still display triadic contacts with presynaptic axons that simultaneously synapse on the afferent bouton and a postsynaptic dendrite [56, 57]. Aδ fibers ending in laminae IIi-III mainly arise from LTMR in hair follicles [58, 59]. A similar pattern of LTMR fibers was described in the caudal Sp5C in the cat [60, 61]. In the rat, large boutons in terminal arbors of myelinated, Aβ fibers, occupy the same layers with a somatotopic pattern [62–64].

The shortest-latency responses to GON electrical stimuli clearly fall within the range of Aβ afferents. Thick fibers with abundant large- and medium-sized terminal and *en passant* boutons are distributed in the lateral one-third of laminae IIi-IV of the upper cervical segments and, more sparsely, along the lateral two-thirds of Sp5C and other lower brain stem structures [27]. Moreover, while the GON stimulation parameters (single pulse, twice the short latency response threshold) are unlikely to recruit unmyelinated C afferents, longer latency responses appearing within the 100 ms interval that mediate the GON and vibrissal stimulations likely represent Aδ fiber activation. Fine myelinated fibers, including Aδ and low conduction velocity Aβ afferents [65] from spinal nerves, distribute mainly in laminae I-IIo and V [66], but may also reach intermediate laminae [67]. Myelinated afferents from GON in laminae II and IV form dense meshworks with terminal and *en passant* boutons of assorted sizes, while those in lamina I mainly consist of long thin axons decorated with abundant *en passant* varicosities, most of them small [27]. In deep laminae, boutons from finely myelinated fibers often make the central element of Type IIa glomeruli, which are postsynaptic to axonal boutons expressing GABA and/or Glycine, as well as GABA-expressing dendrites [53, 54]. In laminae I-IIo these fibers establish simpler axo-dendritic synapses or make the central element of Type I glomeruli, which receive only GABAergic axo-axonic contacts [53, 54].

### TCC neurons driven by vibrissal and/or GON input

All neurons in laminae I-VI had ipsilateral orofacial mechanoreceptive fields, with a predominance of those responding to low-threshold tactile input in laminae III-IV [68]. Laminae IIi-IV of the spino-medullary dorsal horn, where most recordings were made, are the main target for LTMR afferents and a key node for early processing of tactile input. In these laminae in the spinal cord up to seven types of excitatory and four types of inhibitory LTMR interneurons have been described on the basis of molecular-genetic, morphological and electrophysiological profiling [69–72]. These cells account for 98% of all neurons in the region, with a 2.3-to-1 ratio between excitatory and inhibitory cells, and just 2% of ‘projection’ neurons, which send their axons to supraspinal levels through the dorsal or lateral columns [71]. Most, if not all can be monosynaptically driven by primary afferent input to these laminae. Both nociceptive and non-nociceptive input from Aδ afferents reach at least some excitatory (such as PKCγ neurons) and inhibitory (such as islet cells) neurons placed in lamina II and superficial part of III, as well as deeply-placed projection neurons with dendrites extending to superficial laminae [73]. The latter may also show convergence of all kinds of low- and high-threshold afferents [74]. Although comparably less thoroughly investigated, the corresponding laminae in the medullary dorsal horn contain similar neuronal populations and afferent input [75–77].

Although we cannot be sure of the cell type from which we obtained the recordings, indirect data may shed some light on this issue. Spontaneous firing is very low in both WDR and low-threshold mechanosensory-responsive (LTM) neurons of Sp5C under control conditions; following CCI-IoN, however, spontaneous activity markedly increased in the WDR, but not in the LTM [39]. Moreover, it was recently found that excitatory neurons in laminae I-II in rat lumbar spinal cord showed fast adaptation to light tactile stimuli and very low spontaneous firing, whereas inhibitory neurons with a variety of non-adapting responses had much higher spontaneous activity ([78]; see also [38]). Should these findings be applicable to the neurons recorded in TCC, it would indicate that these neurons are likely to be excitatory interneurons or projection neurons.

### Responses in TCC to GON and vibrissal stimulation are differentially affected by CCI-IoN

Injured peripheral nerves exhibit increased ectopic firing, originating in the nerve itself [79] and/or medium-sized neurons in the Aβ and Aδ range in the affected spinal ganglia [80–82]. Both low- and high-threshold mechanosensory ganglion neurons become hyperexcitable and exhibit increased activity as attested by changes in several electrophysiological parameters, and thus may transfer as nociceptive messages normally innocuous tactile stimuli [83]. This abnormal input is responsible for all or most of the increased level of spontaneous activity in dorsal horn WDR neurons, because the conduction block of a constricted nerve proximal to the constriction abolishes it [79]. Nevertheless, the injured nerve causes additional effects, not only bringing about an altered drive on dorsal horn neurons. The sensory neurons affected by nerve lesions also undergo rapid and profound transcription changes [84, 85], so that large afferents change their expression of many transmitters, peptides, and other factors which contribute to drive sensitization and nociceptive responses in DH neurons (reviewed in [86]).

**Fig. 7:**
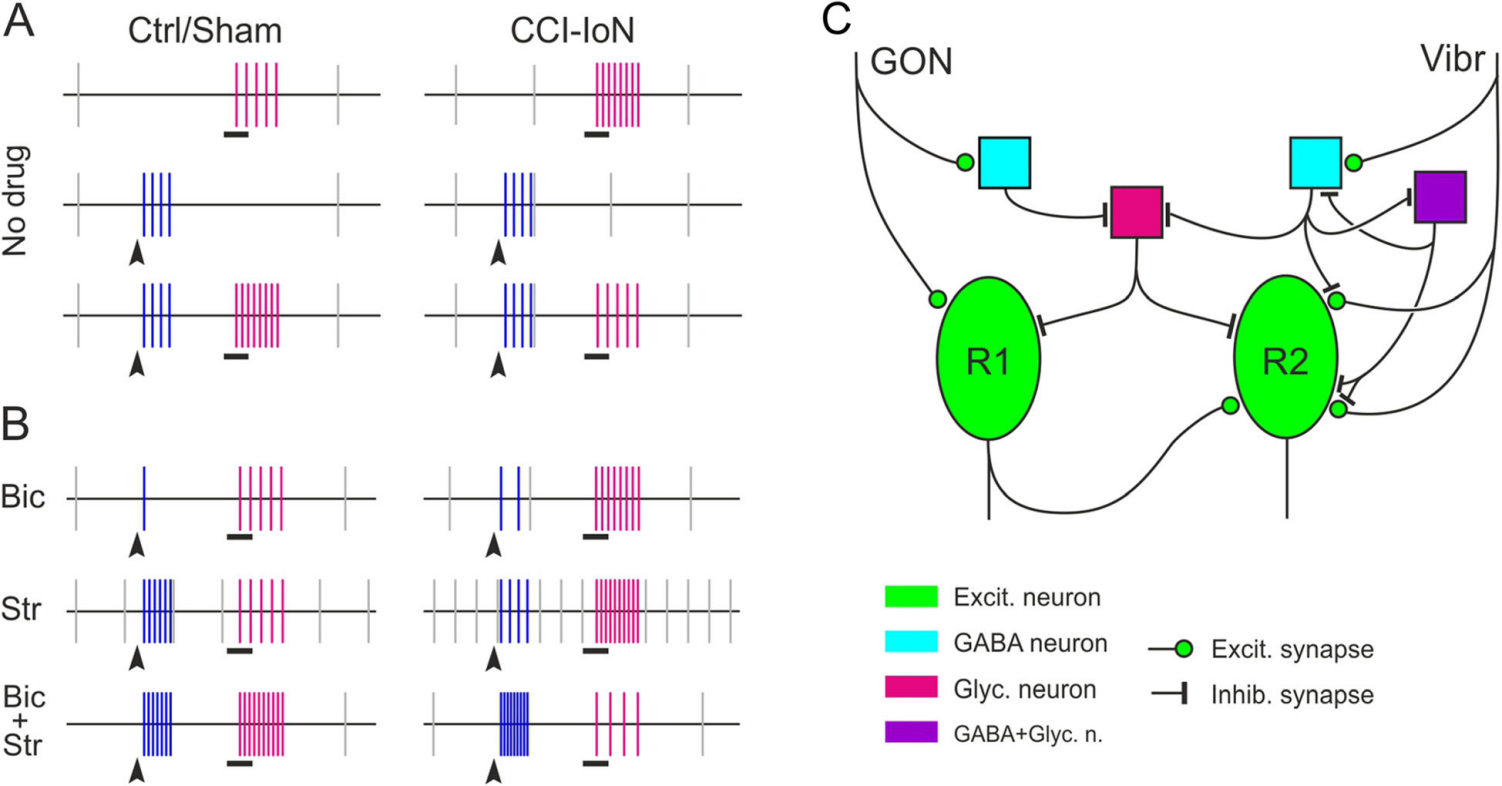
(**a**) Simplified sketch of the neuronal discharges in TCC following vibrissal stimulation (black horizontal bars; magenta vertical bars) and GON stimulation (arrowheads; blue vertical bars). Gray vertical bars indicate the resting, spontaneous discharge of the same TCC units studied after vibrissal stimuli. For better visualization, spontaneous and evoked responses are represented at different time scales (spontaneous activity compressed 10x; interstimulus interval, stimulus duration and firing responses expanded 3-5x). The bottom recordings show the effect of a conditioning GON stimuli on the following response to facial stimuli. (**b**) Similar sketch summarizing the effects of blocking inhibitory GABAergic (Bic), Glycinergic (Str) or both GABA- and Glycinergic transmission (Bic + Str) on the conditioning effect of GON stimulation on the response to vibrissal stimulation. The number of vertical bars under each of these three conditions represent comparisons with the values observed just prior to the application of the drug(s) shown in (**a**). (**c**) Simplified view of a minimal local circuitry in laminae III of TCC that could help explain, at a circuital level only, the effects of GON stimulation on the successive response of TCC units to light stimulation of the vibrissal pad under control or CCI-IoN conditions. Units responding to GON could be different (R1, more likely) or the same (R2, less likely) units recorded after vibrissal stimulation. Color codes and geometric figures identify different excitatory and inhibitory neurons and synapses. Other possible excitatory neurons interposed in the circuit are omitted

In addition to increased spontaneous activity, we found that the response in TCC to light tactile stimulation of the vibrissal pad increased significantly under CCI-IoN, as previously reported for somewhat more rostral levels of Sp5C [39]. A similar finding had also been reported in the lumbar spinal cord after sciatic CCI or spinal nerve ligation [38, 87]. While this increased response could be attributed to both an excess of incoming signals through the IoN and to hyperresponsive neurons in the TCC, it must be noted that such heightened response to peripheral stimuli was not found upon GON stimulation (Fig. 7a). When a spinal nerve is injured, there is an increased expression of peptides involved in central sensitization in small and medium-sized DRG neurons contributing to a neighboring spared spinal nerve [88], as well as electrophysiological features of sensitization in Aβ, Aδ and C nociceptors [52]. These uninjured neurons show a notable overlap of thin afferents in the superficial laminae of the dorsal horn. However, while the GON and trigeminal terminals also show a degree of overlap in the TCC, the corresponding ganglia and peripheral course of these nerves are quite apart and GON ganglia and nerve are undamaged. The fact that responses to GON stimulation did not vary between the controls and CCI-IoN cases suggests that neurons in a TCC sensitized territory only appear hyperresponsive when driven from an injured (IoN) nerve, but not when activated from a converging, but intact (GON) nerve (Fig. 7a). Remarkably, this apparently unchanged response to GON stimulation was ensued by different effects on succeeding responses to facial tactile stimuli depending on whether IoN is, or is not injured, as discussed below.

### A conditioning stimulus to GON increases the TCC response to vibrissal stimulation in controls, but reduces it in CCI-IoN cases

Despite the expanding use of GON stimulation to treat a variety of craniofacial pain disorders (reviewed in [89, 90]), scarce attention has been paid to the basic neural mechanisms that may underlie this connection. Early electrophysiological and anatomical findings, mostly in cats, showed a convergence of GON and trigeminal afferents on upper cervical or medullary dorsal horn (see [27]). More recently, the existence was proved of a functional convergence in Sp5C of nociceptive and non-nociceptive input from different trigeminal domains, such as the supratentorial dura mater and superficial territories of the first and second trigeminal branches [91]), as well as of a direct functional coupling in TCC neurons of dural and cervical afferents conveyed by the GON [26, 28]. However, the interaction between low-threshold afferents and the GON input to TCC, under control and neuropathic conditions, still remained unexplored.

In controls, light vibrissal stimulation elicited a stronger response in the TCC when preceded by a brief electric shock to the GON. This facilitatory effect was due to the activation of NMDA receptors since the effect was blocked by AP-5. Since we could only ascertain that it was the same unit that responded to vibrissal and GON stimulation in some cases, the possibility exists that the GON effects on vibrissal responses are mediated by mono- or polysynaptic connections in the TCC. Those neurons receiving synapses from different sources may show heterosynaptic facilitation, whereby activation of some synapses on a target neuron may potentiate other inactive synapses on the same neuron. This has been demonstrated for low frequency stimulation of C and Aδ fibers in a dorsal root enhancing responses in a motor neuron to input from an adjacent intact root [92], for C fiber stimulation by topical mustard oil unmasking (potentiating) low-threshold A fiber input onto nociceptive-specific and wide dynamic range neurons at superficial or deep dorsal horn laminae [93], or for C or Aδ stimulation-mediated presynaptic potentiation of GABAergic synapses on lamina I neurons [94]. In absence of any direct proof, we think it unlikely that Aδ (or Aβ) input on TCC neurons could potentiate the response to the vibrissal input in the same neurons by a similar mechanism. Moreover, the GON responses in the TCC were all but eliminated by blocking GABA transmission, and enhanced by blocking Glycine transmission (Fig. 7b), suggesting a release of an interposed Glycinergic inhibition probably through a GABAergic neuron, and therefore indicating a predominance of a multisynaptic enhancing effect of the GON activation on the vibrissal responses.

In the CCI-IoN cases, GON stimulation reduced the response to vibrissal stimulation to a level comparable to that seen in the control cases without prior GON stimulation (Fig. 7a). This decrease occurred within a significant increment in the spontaneous activity of the same TCC neurons, consistent with the overall state of GABA- and Glycine-dependent disinhibition in the affected spinal or medullary territory after a neuropathic injury ([39, 95]; see below). Given the essentially unchanged TCC response to GON stimulation compared to controls, it is therefore likely that input from the GON was able to ‘rescue’ local inhibitory circuits that were down-regulated by the nerve injury, thus reducing the TCC response to incoming vibrissal input. To summarize, under CCI, vibrissa-responding TCC units display heightened excitability revealed by their increased basal spontaneous discharges. Hence, GON conditioning is unlikely to affect directly the excitability level of TCC neurons but rather seems to temporally disinhibit local inhibitory GABAergic neurons, thus resetting presynaptic GABAergic and postsynaptic Glycinergic inhibition to normal levels.

### Inhibitory transmission in the TCC and its involvement in the effects of GON stimulation on vibrissal responses

Inhibitory interneurons account for 30–40% of the neurons in laminae I-III of the rat spinal cord, most of which are enriched in GABA, fewer in Glycine, and an undetermined fraction expressing both transmitters in different combinations with other transmitters and neuropeptides [96–98]. In Sp5C about 33% of the neurons express GABA and/or Glycine; of these immunolabeled neurons up to 52% co-express GABA and Glycine, and 17% and 32% express only GABA or only Glycine, respectively [44]. A population of the calcium-binding protein parvalbumin-expressing cells, morphologically assigned to the islet and central types in laminae IIi and dorsal III, co-express GABA and Glycine and have as their predominant synaptic output most of the axo-axonic synapses on boutons from Aβ and Aδ fibers within the same laminae [99, 100].

In addition to exerting presynaptic control on primary afferents, all the inhibitory interneurons make axodendritic and/or axosomatic contacts [71] and, through finely tuned mono- and polysynaptic effects, regulate the transmission of innocuous somatosensory input to other neurons that send both nociceptive and non-nociceptive signals to supraspinal levels (reviewed in [69, 101, 102]. In control conditions, these postsynaptic effects are aimed at controlling the excitability of the dorsal horn neurons, and blocking the flow of excitatory signals to nociceptive-specific projection neurons [103]. Dual simultaneous recordings of synaptically linked interneuron pairs in laminae III-IV have shown that inhibitory synapses outnumber the excitatory ones by 2:1 [104]. In these deeper laminae inhibitory interneurons expressing GAD67 receive predominantly Glycinergic synaptic contacts and are also under tonic extrasynaptic Glycinergic control, in contrast to similar interneurons in laminae I-IIo, which are mostly targets for GABAergic synaptic and extrasynaptic modulation [105]. The importance of Glycinergic inhibition was also found in patch clamp studies on spinal slices, which showed that, although 55% of the neurons in lamina II receive GABA and Glycine synaptic input and all the neurons in laminae III-IV receive GABA and/or Glycinergic input, the inhibitory synaptic transmission is characterized in all cases by a dominant role of Glycinergic inhibition [106]. Moreover, GABA would be acting on presynaptic GABA_B_ receptors, giving a negative feedback signal on the inhibitory afferent (reviewed in [107]). A similar GABA-mediated negative feedback has been shown to occur on glutamate release by Aβ terminals in laminae III-IV [108].

The separate or combined blockade of GABA or Glycine transmission revealed a complex involvement of inhibitory circuits in the effects of GON stimulation on vibrissal responses, and strongly suggests that the synaptic weight of GABAergic and Glycinergic inputs modulating GON and vibrissal inputs is not the same, under either control or CCI-IoN conditions. This complexity was compounded by the uncontrolled degree of penetration of the drugs, a variable in the design intended to mimic the effects of applying agonists or antagonists of inhibitory neurotransmitters intrathecally or intracisternally in both basic research [109–113] and clinical settings [114, 115]. Yet, our findings may be supported by known local connections in the dorsal horn and provide new data on the possible involvement of these circuits under conditions of neuropathic pain.

The GABA_A_ receptor blockade with Bic reduces the response to GON stimulation in both controls and CCI-IoN cases, probably due to a disinhibition of Glycinergic neurons (see above). The effects on the vibrissal responses were negligible in both cases (Fig. 6), but these responses differed when preceded by GON stimulation: The facilitatory effect of GON pre-conditioning shown in controls disappears, consistent with the diminished response to GON stimulus; by contrast, under CCI-IoN, GON stimulation still facilitates the response to the successive facial stimulus, probably because of the local state of disinhibition caused by the CCI [39]. Glycinergic blockade, however, results in a quite different effect on the spontaneous activity, which increases in controls and even more under CCI, and the response to GON, which also increases in controls, but is unaffected by CCI. This would be consistent with the dominant role of Glycinergic inhibition [106], whose removal would disinhibit units responding to the vibrissal input in controls, but would also disinhibit the down-regulated GABAergic transmission under CCI.

As expected, the combined removal of GABA- and Glycinergic transmission produces a marked increase in the responses to the GON and to the vibrissal inputs, but an intriguingly contrasting effect of the conditioning effect of GON stimulus on vibrissal input in controls and in CCI-IoN cases (Fig. 7b). In controls, a strong response to facial stimulation follows the strong response to GON stimulation, consistent with a global state of disinhibition. The heightened response to GON stimulation persists under CCI-IoN, but now the responses to vibrissal stimulation are somewhat decreased by a preceding GON stimulus. Although lacking experimental proof, it may be speculated that the strong response to GON input might have stimulated the release of GABA from local interneurons. With the GABA_A_ receptors blocked by Bic, GABA could still activate GABA_B_ receptors by the extrasynaptic diffusion of the transmitter, not just on a cell postsynaptic to the interneurons, but on other neurons as well [116]. These cells thus become less excitable by the activation of postsynaptic GABA_B_ receptors [117], as will the vibrissal afferents themselves, since presynaptic GABA_B_ receptors are expressed in terminals of Aβ fibers in the spinal laminae III-IV [108]. The circuit diagram sketched in Fig. 7c gives grounds to the proposal that GON-driven alteration of the TCC responses to low-threshold input from the face can be explained, at least in part, by the interplay of inhibitory circuits that modulate the activation of presumed excitatory neurons.

## Conclusion

GON stimulation exerts modulating and opposite effects on TCC neurons in CCI-IoN and control animals. Facilitation was NMDA-dependent while GABAergic and Glycinergic mechanisms mediated inhibition. This work will help us to advance the knowledge of the changes that occur in the synaptic circuitry in the TCC involved in chronic pain, as well as the mechanisms by which stimulating peripheral nerves may bring about beneficial effects on neuropathic pain. A deeper knowledge of these mechanisms will help to identify new therapeutic targets and improve treatments for a number of craniofacial painful conditions.

## Data Availability

The datasets generated for this study are available on request to the corresponding author.

## Abbreviations

AP-5: 2-amino-5-phosphonopentanoic acid
Bic: Bicuculline
CCI-IoN: Chronic constriction injury of the rat infraorbital nerve
CTB: Fraction B of Cholera toxin
DAB: Diaminobenzidine
DC: Dorsal column
dps: Days post-surgery
DRG: Dorsal root ganglion
GABA: Gamma aminobutyric acid
GAD: Glutamic acid decarboxylase
GON: Greater occipital nerve
IB4: Isolectin IB4 from Griffonia simplicifolia
IoN: Infraorbital nerve
NMDA: N-methyl-D-aspartate
LTMR: Low-threshold mechanoreceptor(s)
PKCγ: Protein kinase C gamma
PSTH: Peristimulus time histogram
RF: Receptive field
Sp5C: Caudal division of the spinal trigeminal nucleus
Str: Strychnine
TCC: Trigeminocervical complex
TG: Trigeminal ganglion
WDR: Wide dynamic range
